# Markers Of Glucose Metabolism And Muscle Strength Decline Among The Offspring Of Long-Lived Individuals

**DOI:** 10.1093/geroni/igab046.2330

**Published:** 2021-12-17

**Authors:** Iva Miljkovic, Ryan Cvejkus, Adam Santanasto, Mary Feitosa, Karen Schwander, Mary Wojczynski, Bharat Thyagarajan, Joseph Zmuda

**Affiliations:** 1 University of Pittsburgh, Pittsburgh, Pennsylvania, United States; 2 University of Pittsburgh, Pittsburgh, Pittsburgh, Pennsylvania, United States; 3 Washington University School of Medicine in St. Louis, St. Louis, Missouri, United States; 4 Washington University, St. Louis, Missouri, United States; 5 Washington University School of Medicine, Washington University School of Medicine, Missouri, United States; 6 University of Minnesota, Minneapolis, Minnesota, United States

## Abstract

Diabetes has been linked to accelerated muscle strength decline with aging. However, the association between glucose metabolism and muscle strength decline among individuals without diabetes is less clear. We tested whether fasting plasma markers of glucose and insulin metabolism (glucose, insulin, hemoglobin A1c, and soluble receptor for advanced glycation end products (sRAGE)) are associated with grip strength decline among 1415 non-diabetic offspring of exceptionally long-lived individuals who have a low diabetes risk (age range 36-88; mean age ± SD = 60 ± 8 years; mean BMI ± SD = 27 ± 4.7 kg/m2; 57% women). Grip strength was assessed using a hand-held dynamometer at two clinic visits over an average of 7.9 years. Multiple linear mixed models were adjusted for age, sex, field center, lifestyle, comorbidities, body weight, height, weight change, and family relatedness. Each standard deviation higher fasting insulin (7.3 mIU/L) was related to greater grip strength decline (-0.38 ± 0.16 kg; p=0.016), while each standard deviation higher fasting sRAGE (430 pg/mL) was related to slower grip strength decline (0.36 ± 0.18 kg; p=0.04). Our findings suggest that even among non-diabetic individuals from families with a clustering of “healthier” metabolic profiles - insulin metabolism and advanced glycation end products may be important biomarkers of muscle strength decline with aging. Potential mechanisms, including genetic and metabolic mediators underlying the observed associations, warrant further investigation.

